# Evaluation of Limiting Climatic Factors and Simulation of a Climatically Suitable Habitat for Chinese Sea Buckthorn

**DOI:** 10.1371/journal.pone.0131659

**Published:** 2015-07-15

**Authors:** Guoqing Li, Sheng Du, Ke Guo

**Affiliations:** 1 State Key Laboratory of Soil Erosion and Dryland Farming on the Loess Plateau, Northwest A&F University, Yangling, 712100, China; 2 State Key Laboratory of Vegetation and Environmental Change, Institute of Botany, Chinese Academy of Sciences, Beijing, 100093, China; 3 Institute of Soil and Water Conservation, Chinese Academy of Sciences and Ministry of Water Resources, Yangling, 712100, China; University of New England, AUSTRALIA

## Abstract

Chinese sea buckthorn (*Hippophae rhamnoides* subsp. *sinensis*) has considerable economic potential and plays an important role in reclamation and soil and water conservation. For scientific cultivation of this species across China, we identified the key climatic factors and explored climatically suitable habitat in order to maximize survival of Chinese sea buckthorn using MaxEnt and GIS tools, based on 98 occurrence records from herbarium and publications and 13 climatic factors from Bioclim, Holdridge life zone and Kria' index variables. Our simulation showed that the MaxEnt model performance was significantly better than random, with an average test AUC value of 0.93 with 10-fold cross validation. A jackknife test and the regularized gain change, which were applied to the training algorithm, showed that precipitation of the driest month (PDM), annual precipitation (AP), coldness index (CI) and annual range of temperature (ART) were the most influential climatic factors in limiting the distribution of Chinese sea buckthorn, which explained 70.1% of the variation. The predicted map showed that the core of climatically suitable habitat was distributed from the southwest to northwest of Gansu, Ningxia, Shaanxi and Shanxi provinces, where the most influential climate variables were PDM of 1.0–7.0 mm, AP of 344.0–1089.0 mm, CI of -47.7–0.0°C, and ART of 26.1–45.0°C. We conclude that the distribution patterns of Chinese sea buckthorn are related to the northwest winter monsoon, the southwest summer monsoon and the southeast summer monsoon systems in China.

## Introduction

Chinese sea buckthorn (*Hippophae rhamnoides* subsp. *Sinensis*) is a subspecies that belongs to the family Elaeagnaceae [1,2], and is also commonly called acid vinegar salix or black thorn. It has the largest distribution and planting area of all species belonged to the same genus, and it is the most important economic and ecological shrub species. Thus far, the ecological effects of Chinese sea buckthorn have been well documented. It improves soil properties, reduces pollution and prevents soil erosion. Economically, the plant species has not only been considered as an ornamental shrub but also been used as a source of nutritious food, medicine and firewood [3–5]. Therefore, Chinese sea buckthorn has been arousing international attention as a promising new crop, and it is predicted by some as the next major health food fad [3]. Since 1985, Chinese sea buckthorn has been used to control accelerated desertification under an initiative of the government and the cultivation of this species has been widely promoted in northwest China. In the past 20 years, the planting area of Chinese sea buckthorn has reached approximately 14,667.4 km^2^ (22 million acres) [6], and increased by more than 800 km^2^ annually [7]. Detailed information of ecological characteristics, suitable habitat and potential distribution of Chinese sea buckthorn is needed for improvement of scientific research and general cultivation of this plant species.

Climate has been documented to play a critical role in determining large-scale species distributions [8–11]. Climatic factors are considered effective surrogates for resource needs or other limiting factors, which may have a stronger forecast ability than biophysical factors in predicting species distribution and growth [12,13]. Gu et al. [14] studied the factors that determined the distribution of Chinese sea buckthorn on the Tibetan Plateau and found that precipitation was the key factor affecting distribution of the plant species. Lian and Chen [15] believed that Chinese sea buckthorn has a stable distribution area, which could be used as an indicator for China's three major vegetation zone boundaries (forest, steppe and alpine zones). These studies greatly improve our understanding of the influence of climatic factors on geographic distribution of Chinese sea buckthorn. However, these studies are based on empirical data, and they failed to provide/quantify the relative importance of climatic factors, response curves and climatic thresholds of this species.

The current dominant method to explore climate-species distributions is using species distribution models (SDMs), which is based on species niche theory [16–19]. Several methods are available to simulate species distribution patterns [20,21], and these models can be classified into mechanistic modeling approaches and statistical modeling approaches. Mechanistic modeling approaches mainly simulate the fundamental niche of a species, while statistical modelling approaches investigate the realized niche of a species [22,23]. Mechanistic modeling approaches, such as the CLIMEX model [24–29], have been used extensively to simulate various species of agricultural and non-agricultural crops as well as pests. But Mechanistic modeling approaches are based on many eco-physiological parameters, which are rarely obtained for a majority of species, including Chinese sea buckthorn. Statistical modeling approaches can be classified into two groups: those requiring presence-absence data and those requiring presence-only data [22,30,31]. The latter group has been developed recently and is gaining popularity because of the availability of presence-only data. Financial constraints, difficulty in conducting field inventories and the lack of reliable or meaningful absence data has also made the models requiring presence-only data more appealing (e.g. genetic algorithms [32], maximum entropy approach [33,34], bioclimatic envelope [35] and distance algorithms [36]). Such modeling techniques are recommended when absence data of a species is not available or reliable [30,37]. Although there is high consistency between models, many studies have shown that maximum entropy modeling (MaxEnt) is widely used and usually produces accurate predictions of species distributions [20,21,38]. Furthermore, Elith et al. [39] expanded the ability of the MaxEnt model by increasing the limiting factor mapping and similar surface mapping for range-shifting species, which is especially suitable for predicting potential species distributions and interpretation of limiting factors.

The purpose of our study was to examine how climate factors limit the distribution of Chinese sea buckthorn and to simulate the climatically suitable habitat for Chinese sea buckthorn by utilizing the available data of species occurrence, features of the MaxEnt model and a geographic information system (GIS). Our study mainly focuses on the following objectives: (1) to identify the important climatic factors and where the limiting factors affect the distribution boundaries of this species; (2) to identify the climatic thresholds and find out the climatically suitable habitats for Chinese sea buckthorn; and (3) to explain the distribution patterns based on the perspective of East Asian monsoon climate. Our study could provide theoretical support for top-level design of the cultivation and planting of Chinese sea buckthorn for policy-makers and planners.

## Materials and Methods

### Study area and species records

**Fig 1:**
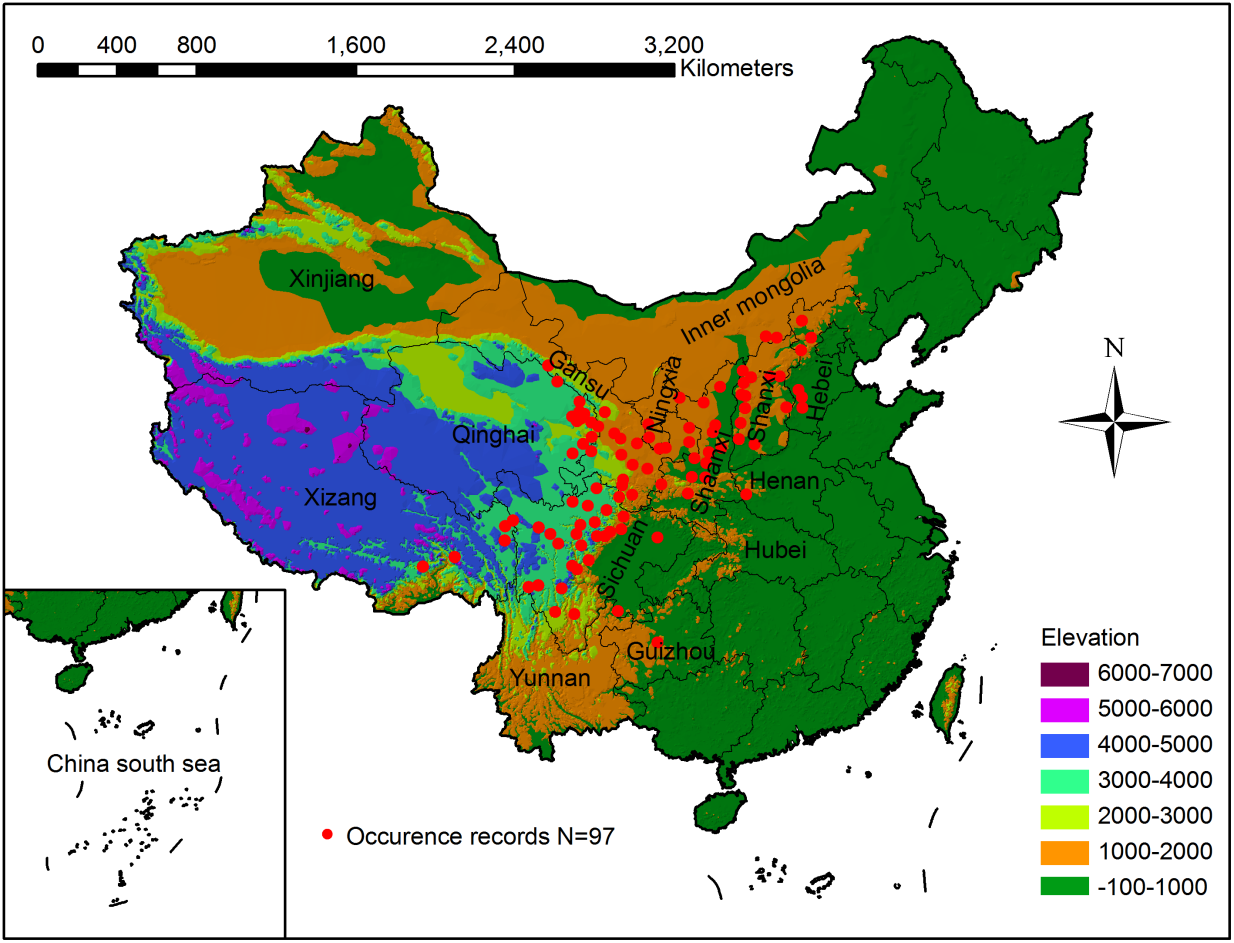
Occurrence records of Chinese sea buckthorn with topography in China. Records were collected from herbariums and published sources, providing 97 occurrence points.

The study was conducted in China, which is located in the eastern part of Asia, on the western coast of the Pacific Ocean (3°52′–53°33′N, 73°40′–135°2′E), and spans are over 5,000 km from east to west and over 5,500 km from north to south. The summer monsoons dominate the climate of the country, and the topography descends in a three-step staircase from west to east (Fig 1). China covers a land area of about 9.6 million km^2^, occupying one-fifteenth of the world’s total land area. Because of its large area and extent, as well as its high population, China has a wide variety of climates, topography, soil and vegetation types [40].

Species records of Chinese sea buckthorn in continental China were mainly collected from published resources [14,15,41] and the Chinese Virtual Herbarium [42], which integrates herbarium data of national natural museums from 14 institutes in China. We found 368 specimens, and then removed duplicate specimens and specimens with no location/coordinates information. The longitude and latitude of the records were accurate at the county level. Consequently, 97 records were collected from all sources. Spatial distribution pattern of 97 records relating to topography of China can be seen in Fig 1 and latitude/longitude coordinates of each record was stored in an Excel database (S1 File) for MaxEnt model building.

### Climatic variables

Many climatic variables have been used to characterize hydrological-thermal climatic niches in China, such as BIOCLIM variables [43,44], Holdridge life zone variables [8] and Kira's index variables [45,46]. These variables have been widely used in research on the relationship between species/vegetation and climate at a regional or global scale. The BIOCLIM variables have been widely used in SDMs studies, as the data can be easily downloaded from the WorldClim database with no further calculation requirement. Holdridge life zone and Kira's index variables are seldom used in SDMs studies, but they still provide considerable power for explaining species distributions. Here, we integrated these climatic variables and selected 13 climatic variables (Table 1), including 5 variables from Holdridge life zone and Kira's index variables and 8 variables from Bioclim variables, mainly by reducing redundant information in Bioclim variables and considering extreme climatic variables that usually limit species distribution [47]. Baseline climatic layers were downloaded from the WorldClim database at a spatial resolution of 10 arc-min, which were generated by using thin-plate smoothing splines with latitude, longitude, altitude, and monthly temperature and precipitation records from 1950–2000 from climate stations [43,44].

**Table 1:** Description of 13 climatic factors and corresponding calculated formula.

Variable	Abbreviation	Unit
**Annual mean temperature**	AMT	°C
**Mean temperature of the warmest month**	MTWM	°C
**Mean temperature of the coldest month**	MTCM	°C
**Annual range of temperature** ^**a**^	ART	°C
**Annual precipitation**	AP	mm
**Precipitation of wettest month**	PWM	mm
**Precipitation of driest month**	PDM	mm
**Precipitation of seasonality** ^**b**^	PSD	%
**Annual biotemperature** ^**c**^	ABT	°C
**Warmth index** ^**d**^	WI	°C
**Coldness index** ^**e**^	CI	°C
**Potential evapotranspiration rate** ^**f**^	PER	°C /mm
**Humidity index** ^g^	HI	mm/°C

^a^ ART = Mean of monthly (max temp—min temp).

^b^ PSD = Coefficient of variation.

^c^ ABT = (∑T)/12 (T is 0<T<30°C mean month temperature).

^d^ WI = ∑(T-5) (T is >5°C mean month temperature).

^e^ CI = -∑(5-T) (T is <5°C mean month temperature).

^f^ PER = 58.93×ABT/AP;

^g^ HI = AP/WI.

### Model selection and evaluation

This study utilized MaxEnt software (version 3.3), a machine-learning algorithm designed by Phillips et al. [33,34,48]. MaxEnt expresses the suitability of a grid cell as a function of the environmental or climate layers at that grid cell in a landscape, together with a set of sample locations where the plant species has been observed. The distribution of suitability is proved to be the same as the Gibbs distribution that maximizes the product of the probabilities of the sample locations, where the Gibbs distribution takes the form (Eq 1):

$$P(x)=exp(c_{1}\times f_{1}(x)+c_{2}\times f_{2}(x)+c_{3}\times f_{3}(x)\ldots )/Z$$ (1)

Here *c*_1_, *c*_2_, *c*_3_,… are constants, *f*_1_, *f*_2_, *f*_3_,… are the features, and *Z* is a scaling constant that ensures that *P* sums to 1 over all grid cells.

Elith *et al*. [39] enhanced the ability to explain the MaxEnt model outputs by integrating multivariate environmental similarity surface (MESS) analysis and limiting factors mapping in the new version of MaxEnt (3.3). MESS analysis assists in revealing whether there is possible model-predicted novel habitat (extrapolation), which informs the credibility of model output. If we let min_*i*_ be the minimum value of variable *V*_*i*_ over the reference point set and the similarly for max_*i*_, *p*_*i*_ be the value of variable *v*_*i*_ at point *P*, *f*_*i*_ be the percent of reference points whose value of variable *V*_*i*_ is smaller than *p*_*i*_, then the similarity of *P* with respect to variable *V*_*i*_ is calculated by Eq 2:

$$\{\begin{array}{ll} \left(p_{i}-{\text{min}}_{i})/({\text{max}}_{i}-{\text{min}}_{i}\right) & if\;f_{i}=0\\ 2\times f_{i} & if\;0<f_{i}\leq 50\\ 2\times (100-f_{i}) & if\;50<f_{i}<100\\ ({\text{max}}_{i}-p_{i})/({\text{max}}_{i}-{\text{min}}_{i})\times 100 & if\;f_{i}=100 \end{array}$$ (2)

Finally, the multivariate similarity of *P* is the minimum of its similarity with respect to each variable. The MESS is similar to a BIOCLIM analysis [35] that makes use of percentiles, but it is extended to give negative values so as to differentiate levels of dissimilarity when outside the range of the reference points, where the reliability of model output depends on the accuracy of species response curves. MESS results allow the mapping of locations where limiting factors are important. The assumption is that information on which variable is driving the MESS value at any given point can be extracted and mapped by finding the most dissimilar variable. The most dissimilar variable for a point *P* is the variable with the smallest value of similarity to *P* [39]. According to map of limiting factors, we could easily deduce which factors are limiting physiological and ecological processes.

The MaxEnt model is usually evaluated by the threshold-independent receiver operating characteristic (ROC) approach [calculating the area under the ROC curve (AUC) as a measure of prediction success], which is a graphical method that represents the relationship between the false-positive fraction (1-specificity) and the sensitivity for a range of thresholds [49]. It has a range of 0–1, with a value greater than 0.5 indicating a better-than-random performance event. A rough classification guide is the traditional academic point system [50]: poor (0.5–0.6), fair (0.6–0.7), good (0.7–0.8), very good (0.8–0.9), and excellent (0.9–1.0).

### Simulating process and statistical analysis

Coordinates of the Chinese sea buckthorn presence points were uploaded into the MaxEnt software, together with all of the 13 climatic variables. Linear, quadratic, product, threshold, and hinge features were used to generate feature types. A linear feature is simply one of the continuous environmental variables. A quadratic feature is the square of one of the continuous environmental variables. A product feature is the product of two continuous environmental variables; when used with linear and quadratic features, product features constrain the output distributions to have the same covariance for each pair of environmental variables as the samples. A threshold feature is derived from a continuous environmental variable. For a threshold value *v*, the threshold feature is binary (taking values 0 and 1) and is 1 when the variable has value greater than *v*. A hinge feature is also derived from a continuous environmental variable. It is like a linear feature, but it is constant below a threshold *v*. The convergence threshold (10^−5^), maximum number of iterations (500), and 10,000 global background points were used to run the MaxEnt model. The logistic output (scaled up in a non-linear manner) was used to estimate the probability of presence (ranging from 0–1), which was easier to use and interpret than the other two outputs (raw and cumulative formats).

A jackknife test (systematically leaving out each variable) and the regularized gain change [log of the number of grid cells minus the log loss (average of the negative log probabilities of the sample locations)] were then used to evaluate which climatic factors were the most important in determining the potential distribution of the species. Response curves were created by using the MaxEnt model only with the corresponding variable to avoid the potential problem of strongly correlated factors on the explanation of species response curves. MESS analysis and the limiting mapping technique were used to determine the location of novel habitat and where the limiting factors affected the distribution boundary of the species (each reference point indicates the occurrence records for the species).

Considering the large amount of occurrence data, we used a 10-fold cross-validation (sometimes called rotation estimation) method to evaluate the performance of the MaxEnt model. This method randomly partitioned the original sample into 10 subsamples of equal size. Of the 10 subsamples, 1 subsample was used as testing data, while the remaining 9 subsamples were used as training data. The cross-validation was then repeated 10- fold, and each observation was used for validation exactly once. The AUC values produced from the 10-fold cross-validation were then averaged to indicate the performance of the MaxEnt model. Cross-validation is important in testing model performance, especially where further sample collections are hazardous, costly, or impossible to collect.

A suitable habitat map for Chinese sea buckthorn was produced by utilizing the AUC weight averages of the 10 logistic output maps produced by 10-fold cross-validation, in which the relative suitability range was from 0 to 1. Normally, a threshold of 0.5 is set, which means that a grid cell with a value equal to or greater than 0.5 is recorded as presence, while a value smaller than 0.5 is recorded as absence. In order to retain the maximum predictive information, we divided the predicted suitability into 4 habitat class levels: core area (0.75–1.00), moderately suitable area (0.50–0.75), marginal area (0.25–0.50), and unsuitable area (0.00–0.25). The climatic thresholds (conditions) for each habitat class were analyzed using a GIS tool by ArcGIS 9.3 [51].

## Results

### Current and potential distribution of Chinese sea buckthorn

**Fig 2:**
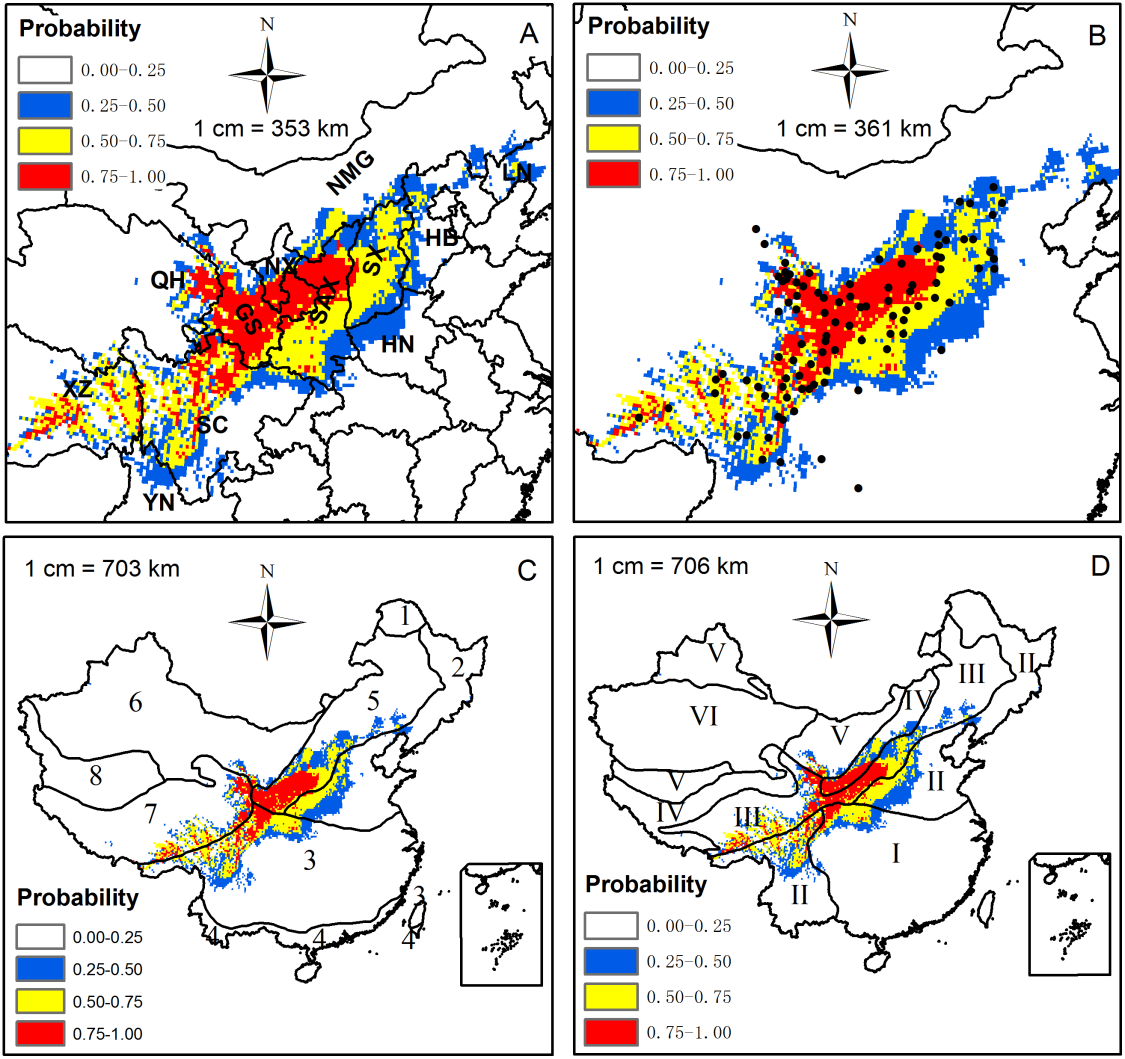
Potential distribution of Chinese sea buckthorn in China and its relationships with (A) Individual provinces, (B) Occurrence records, (C) Vegetation divisions, and (D) Dry & wet divisions. XZ (Xizang), YN (Yunnan), SC (Sichuan), QH (Qinghai), GS (Gansu), NX (Ningxia), SAX (Shaanxi), SX (Shanxi), HN (Henan), HB (Hebei), NMG (Inner Mongolia), LN (Liaoning). 1) Cold temperate deciduous needle-leaved forest region; 2) Temperate deciduous broad-leaved forest region; 3) Subtropical evergreen broad leaved forest region; 4) Tropical seasonal rain forest region; 5) Temperate steppe region; 6) Temperate desert region; 7) Alpine meadow and steppe region; 8) Alpine semi-desert and desert region. I) Humid region with non-distinct dry season; II) Humid region with distinct dry season; III) Semi-humid region; IV) Semi-arid region; V) Arid region; VI) Extremely arid region.

According to the locations of Chinese sea buckthorn occurrence records, a map showing current distribution of this plant species was produced (Fig 1). Chinese sea buckthorn mainly occurs in seven provinces: Xizang, Sichuan, Qinghai, Gansu, Ningxia, Shaanxi and Shanxi. When the maps obtained from the 10-fold cross-validation were AUC weight averaged, a climatically suitable habitat map for Chinese sea buckthorn was created in Fig 2. The potential distribution of Chinese sea buckthorn is mainly located in semi-humid and semi-arid regions (Fig 2D), and begins from southwest of the Hengduanshan Mountains and extends to the southern boundary of the Daxinganling Mountains (extending southwest to northeast at 45°). The altitude decreases from the southwest (about 3500–4100 m) in the Hengduanshan Mountain region to northeast (1000–1500 m) in the Loess Plateau region. The width of the range is about 560 km with an area of approximately 1.12 million km^2^, occupying 11.7% of the land area of China, spanning 12 provinces including Xizang, Yunnan, Sichuan, Gansu, Qinghai, Ningxia, Shaanxi, Shanxi, Hebei, Inner Mongolia, Henan, and Liaoning with the core areas mainly located in Xizang, Sichuan, Gansu, Qinghai, Ningxia and Shaanxi provinces (Fig 2A). The distribution is located in the transition zone of China’s three major vegetation zones (forest, steppe and alpine zones), primarily in the transitions between subtropical evergreen broadleaf forest region to alpine meadow and steppe region, and temperate deciduous broad-leaved forest region to the temperate steppe region (Fig 2C) [40,52].

Based on the suitability level map of Chinese sea buckthorn simulated by MaxEnt (Fig 2), Four habitat categories were defined: the core area (0.75 to 1.00), the moderately suitable area (0.50 to 0.75), the marginal area (0.25 to 0.50), and the unsuitable area (0.00 to 0.25). The climatic thresholds for the habitat categories are shown in Table 2. The results show that the climatic thresholds for the core areas of Chinese sea buckthorn are as follows: PDM of 1.0–7.0 mm, AP of 344.0–1089.0 mm, CI of -47.7–0.0°C, ART of 26.1–45.0°C, PWM of 81.0–230.0 mm, WI of 42.0–170.4°C, PSD of 72.0–106.0 mm, and MTCM of -19.8– -1.3°C.

**Table 2:** Climatic threshold of suitable habitat map for Chinese sea buckthorn predicted by the MaxEnt model.

Climatic Factor	Relative Importance %	Climatic Suitable Habitat Map			
		Core Area 0.75–1.00	Medium Area 0.50–0.75	Marginal Area 0.25–0.50	Unsuitable Area 0.00–0.25
**PDM**	21.1	1.0–7.0	0.0–10.0	0.0–17.0	0.0–192.0
**AP**	20.4	344.0–1089.0	281.0–1226.0	217.0–1599.0	14.0–4000.0
**CI**	16.2	-44.7–0.0	-48.1–0.0	-57.5–0.0	-164.2–0.0
**ART**	12.4	26.1–45.0	25.9–45.0	23.0–47.9	14.1–61.2
**PWM**	7.9	81.0–230.0	77.0–264.0	51.0–341.0	3.0–941.0
**WI**	6.5	42.0–170.4	35.1–181.0	26.6–208.4	0.0–300.6
**PSD**	6.2	72.0–106.0	68.0–112.0	61.0–141.0	19.0–151.0
**MTCM**	4.3	-19.8– -1.3	-22.1–0.1	-23.5–2.7	-38.9–17.2
**MTWM**	1.8	16.2–33.4	13.2–34.9	11.8–34.5	5.4–41.1
**HI**	1.1	3.0–21.0	2.5–28.5	2.3–37.1	0.0–97.7
**AMT**	1.0	0.9–14.7	-0.4–15.5	-1.3–17.8	-13.2–25.5
**PER**	1.0	0.2–1.6	0.1–1.9	0.1–2.1	0.0–63.9
**ABT**	0.1	3.7–14.7	3.2–15.5	2.4–18.7	0.0–25.5

The abbreviation of climatic factors and their units can be seen in Table 1.

### Model performance and importance of climatic factors

**Fig 3:**
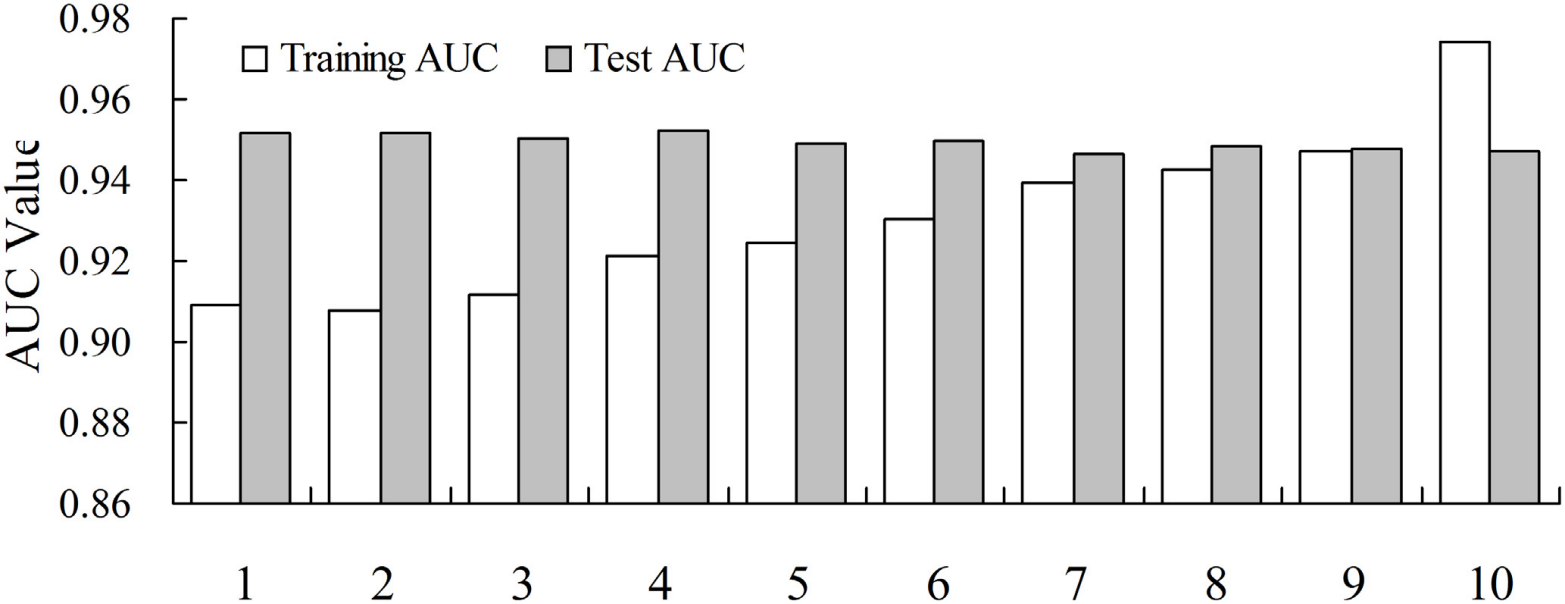
Areas under the receiver operating characteristic curve (AUC) value of test data and training data based on 10-fold cross validation ascending in order by AUC value. X-axis label 1–10 represents the model code. The mean test AUC value is 0.93 and the mean training AUC value is 0.95.

The 10-fold cross-validation method and areas under the receiver operating characteristic curve method were used to assess the accuracy of the MaxEnt model. The AUC values of test data and training data based on 10-fold cross validation in ascending order by training AUC value were shown in Fig 3, which indicated that the MaxEnt model performance was highly accurate (AUC value greater than 0.9), with an average test AUC value of 0.93 (0.9077 to 0.9745) and an average training AUC value of 0.95 (0.9471 to 0.9523). The coefficient of variation in test AUC values was only 2.2% among 10 model simulations, which showed that the 10-fold cross-validation method didn't influence the predicted performance of the MaxEnt model.

**Fig 4:**
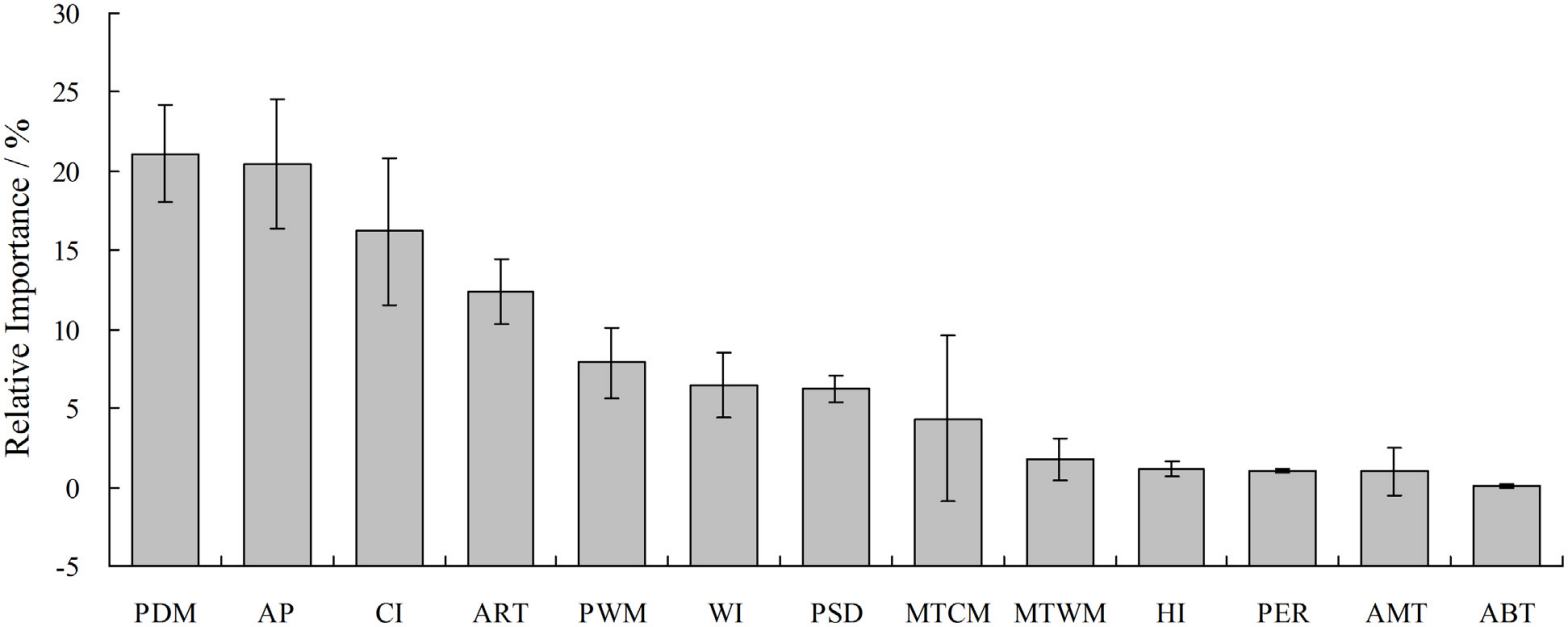
The relative importance of climatic factors in limiting the potential distribution of Chinese sea buckthorn. The meanings of abbreviations of PDM, AP, CI, ART, PWM, WI, PSD, MTCM, MTWM, HI, PER, AMT, and ABT can be found in Table 1.

The relative contribution of climatic variables (in descending order) in determining the potential distribution of Chinese sea buckthorn was shown in Fig 4. The results indicated that PDM, AP, CI and ART were the most influential climatic factors in limiting the potential distribution of Chinese sea buckthorn and these 4 factors explained 70.1% of the variation (12.4–21.1% for each factor), followed by PWM, WI, PSD and MTCM, which explained another 24.9% of the variation (4.3–7.9% for each factor). The remaining 5 climatic factors were unimportant in limiting the distribution of the species, and they accounted for 5% of the variation (0.1–1.8% for each factor). These significant factors can be divided into hydrological-related factors (PDM, AP, PWM and PSD, accounting for 55.6% of the variance) and thermal-related factors (CI, ART, WI and MTCM, accounting for 39.4% of the variance). The results indicated that hydrological related climatic factors played a more important role than thermal related climatic factors in controlling the potential distribution of Chinese sea buckthorn in China.

**Fig 5:**
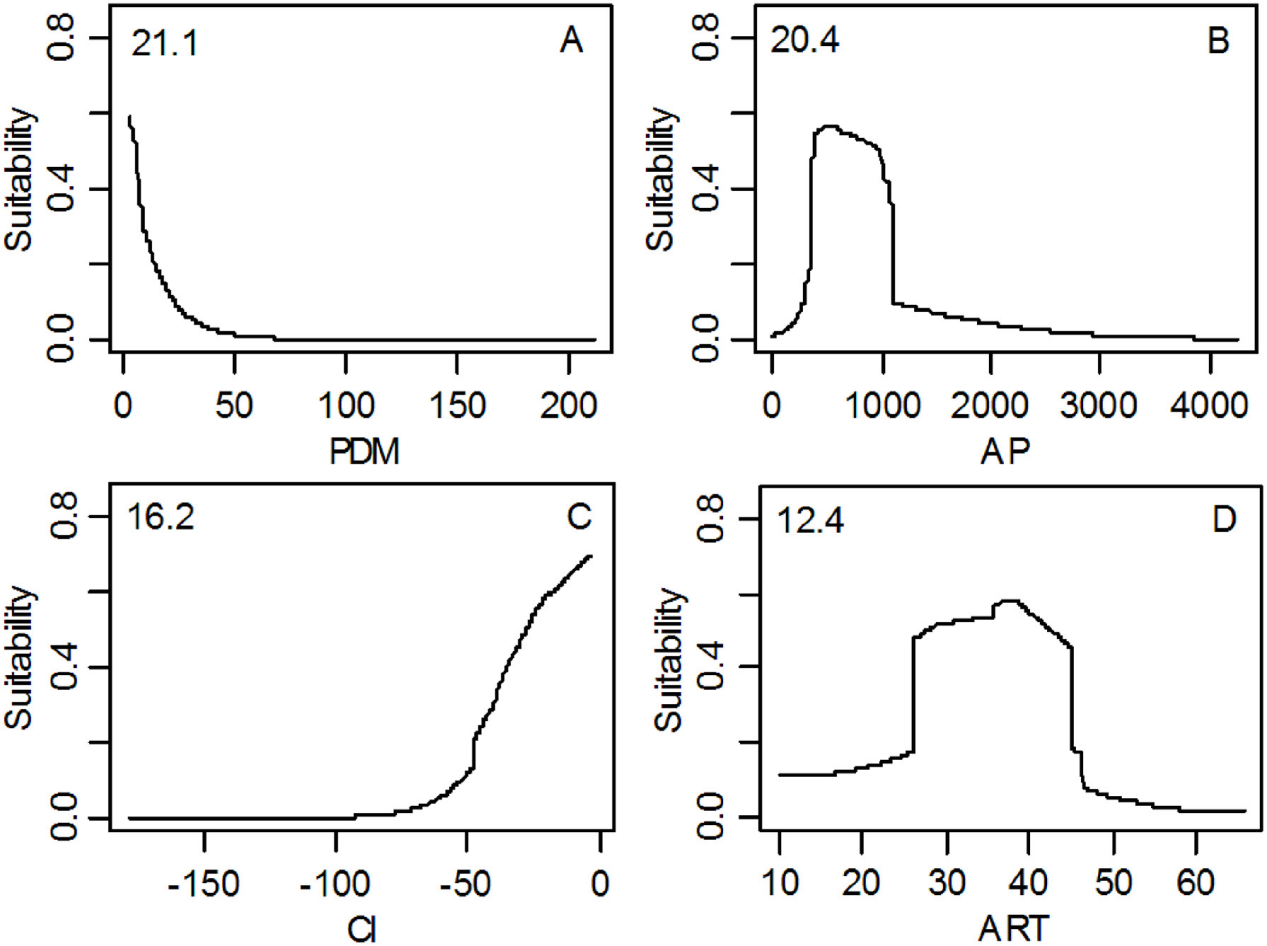
The response curve of climatic suitability for four dominant climatic factors and their relative importance showing in the upper-left corner of each subplot. (A) Precipitation of the driest month (PDM, mm); (B) Annual precipitation (AP, mm); (C) Coldness index (CI, °C); (D) Annual range of temperature (ART, °C); Exponential response curve (PDM, CI); Unimodal response curve (AP, ART).

Response curves to climatic suitability for all climatic factors were shown in S1 Fig Upward trends for variables indicated a positive relationship, while downward trends represented a negative relationship. The magnitude of these trends indicated the strength of the relationship. The four most influential climatic factors (PDM, AP, CI and ART) were shown in Fig 5. The results clearly illustrated that PDM was best described by exponential decay and CI showed an exponential increase relationship, whereas AP and ART showed a bell curve relationship. Response peaks to habitat suitability for PDM, AP, CI and ART were 0 mm, 540 mm, 0°C and 38°C, respectively.

## Discussion

**Fig 6:**
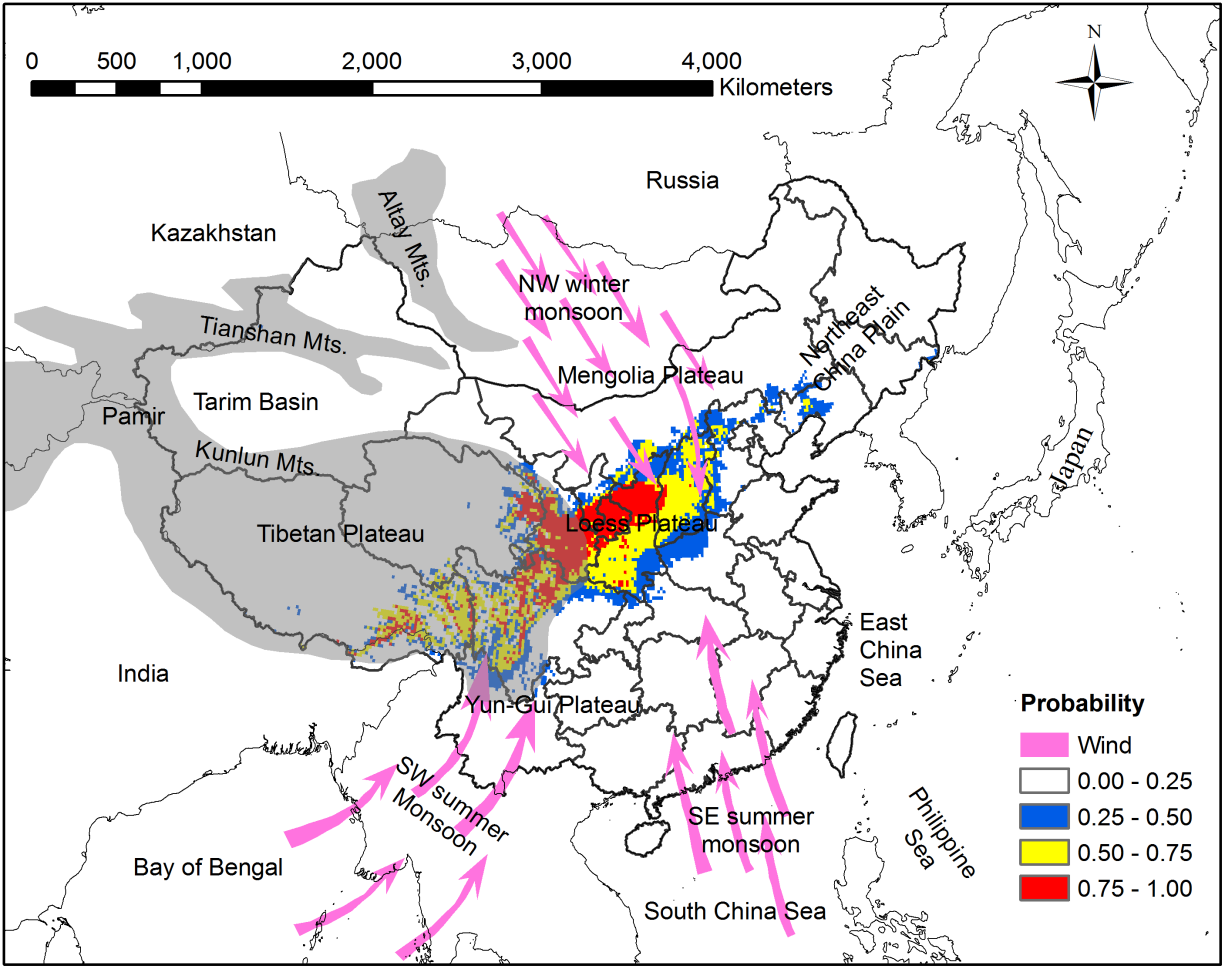
Multivariate environmental similarity surface (MESS) map of novel habitat. Coarse blue polygon represents potential distribution range of Chinese sea buckthorn using threshold of 0.5. Red color represents interpolation habitat (positive value), green color represents extrapolation habitat (negative value), and orange represents marginal habitat (near zero).

SDMs have become the subject of an active field of research in large-scale ecology and biogeography, and they have been used to solve many ecological issues in recent decades [19,22,23]. These models have been used for biodiversity assessment, biological reserve design, habitat management and restoration, invasive species and pest threat management, species introduction and cultivation, and predicting the effects of climate change on species distributions [53–59]. The assumption underlying the SDMs is that species distribution is in equilibrium with climatic conditions and that the sample records used for the training model are sufficient and they represent the niche of the species. In this study, the potential distribution of Chinese sea buckthorn was found to be similar to its occurrence records (Fig 2B), which demonstrates that the distribution pattern of Chinese sea buckthorn is in equilibrium with climatic conditions. The results of MaxEnt model showed that little novel habitat outside the blue polygon as shown in the MESS map (Fig 6), which indicates that the MaxEnt model output had high reliability, because the habitat was interpolated, but not extrapolated, by the MaxEnt algorithm. Therefore, the results demonstrated that the occurrence records were sufficient to simulate the MaxEnt model, which was further shown by the excellent AUC performance with 0.93 (significantly better than random). In addition, the MaxEnt model is an open source and free software, and the workflow integrating MaxEnt and GIS tools used in this study are ideal for other species around the world.

**Fig 7:**
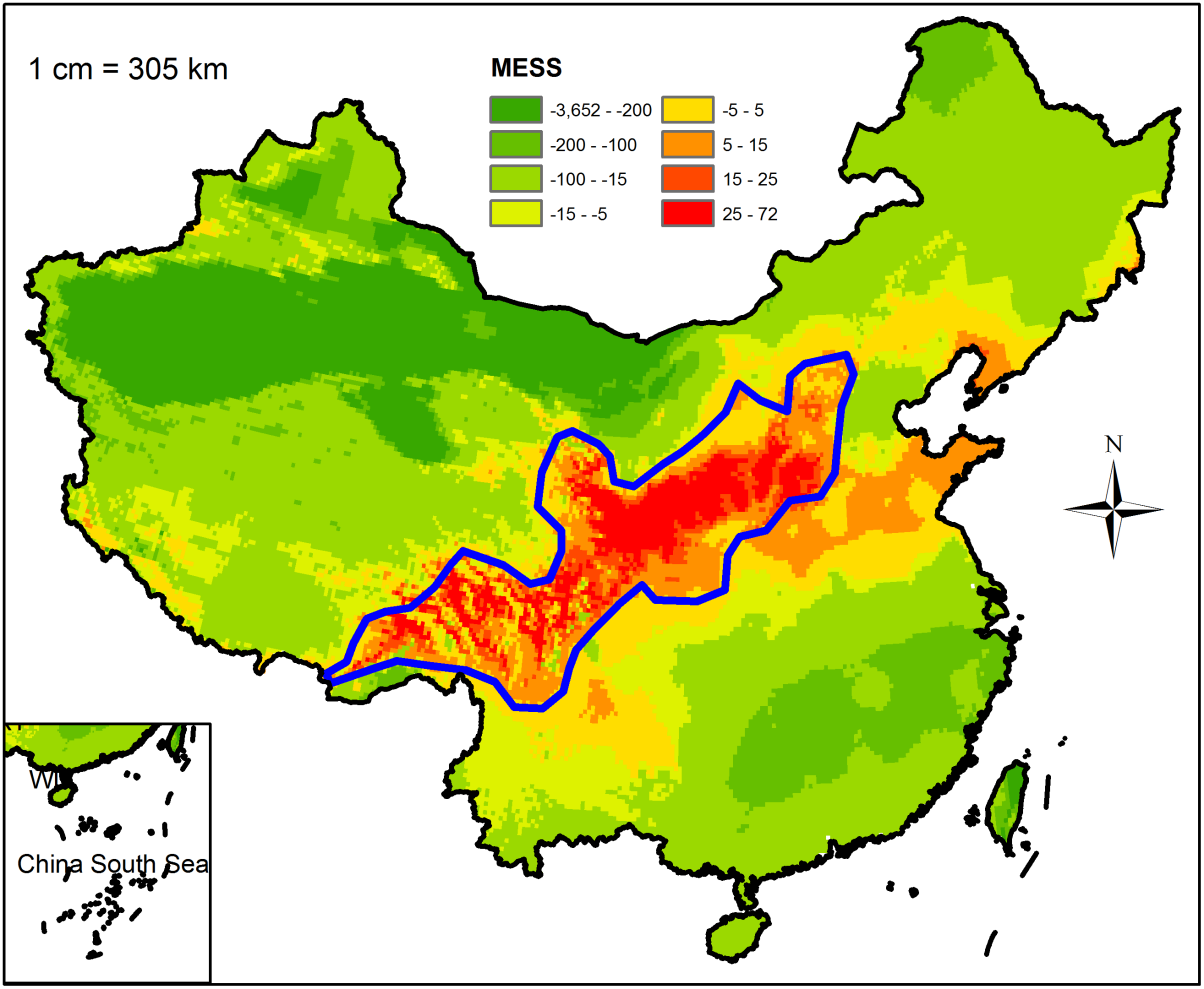
Distribution pattern of Chinese sea buckthorn, monsoon climate and four Plateaus (Tibetan Plateau, Yun-Gui Plateau, Loess Plateau, and Mengolia Plateau) in China. Grey portion represents high altitude region including Tibetan Plateau, Pamir Plateau, Tianshan Mts., and Altay Mts. Monsoon climate includes the northwest winter monsoon, the southwest summer monsoon, and the southeast summer monsoon.

The MaxEnt model shows that the potential distribution pattern of Chinese sea buckthorn (southwest to northeast orientation) is perpendicular to the East Asian monsoon sweep direction (southeast to northwest orientation) (Fig 7) [60]. We speculated that the distribution patterns were related to the northwest winter monsoon, the southwest summer monsoon, and the southeast summer monsoon systems in China. In winter, the Siberian cold winter monsoon blows from the northwest to southeast, which brings dry and cold air. In summer, the southeast summer monsoon blows from the Pacific Ocean and the southwest summer monsoon blows from the Indian Ocean, which brings moist and warm air [61]. In the northwest of potential distribution area of Chinese sea buckthorn, the plant species mainly locates in the center of the Loess Plateau, which belongs to inland area of China and is far away from East China Sea. Therefore, air mass of Eastern Asian monsoon arrives in Loess Plateau later and continues for shorter period, while that of Siberian cold winter monsoon reaches this area earlier and lasts for longer time within a year [62,63]. In the southwest of potential distribution area of Chinese sea buckthorn, the plant species mainly locates in the Hengduanshan Mountains, where the climate is mainly influenced by the southwest monsoon and Tibetan Plateau (the roof of the world) [60]. From Yun-Gui Plateau to Tibetan Plateau, there are many dry-hot valleys of north-south orientation across complex terrain conditions (Fig 1), which are the suitable habitats for Chinese sea buckthorn (Figs 1 and 7). When the southwest monsoon blows from Yun-Gui Plateau to Tibetan Plateau in summer, the warm and moist air mass can only sweeps northward through the north-south valleys. The distribution pattern of Chinese sea buckthorn is very similar to that of liaotung oak (*Quercus wutaishannica*), a species endemic and native to China [64]. However, it is worth mentioning that the south boundary of liaotung oak is not able to extend to the northern slope of the Qinling Mountains, which represent a dividing line between the warm temperate and northern subtropical zone in China. The exotic species black locust (*Robinia pseudoacacia*) could survive in the same region as Chinese sea buckthorn, but the distribution of black locust does not show the southwest-northeast direction [47] because black locust is mainly limited by low temperatures unlike those of the Eastern Asia monsoon climate. Accordingly, we speculated that other native species in this region are influenced by monsoon climate, but further verification is necessary.

**Fig 8:**
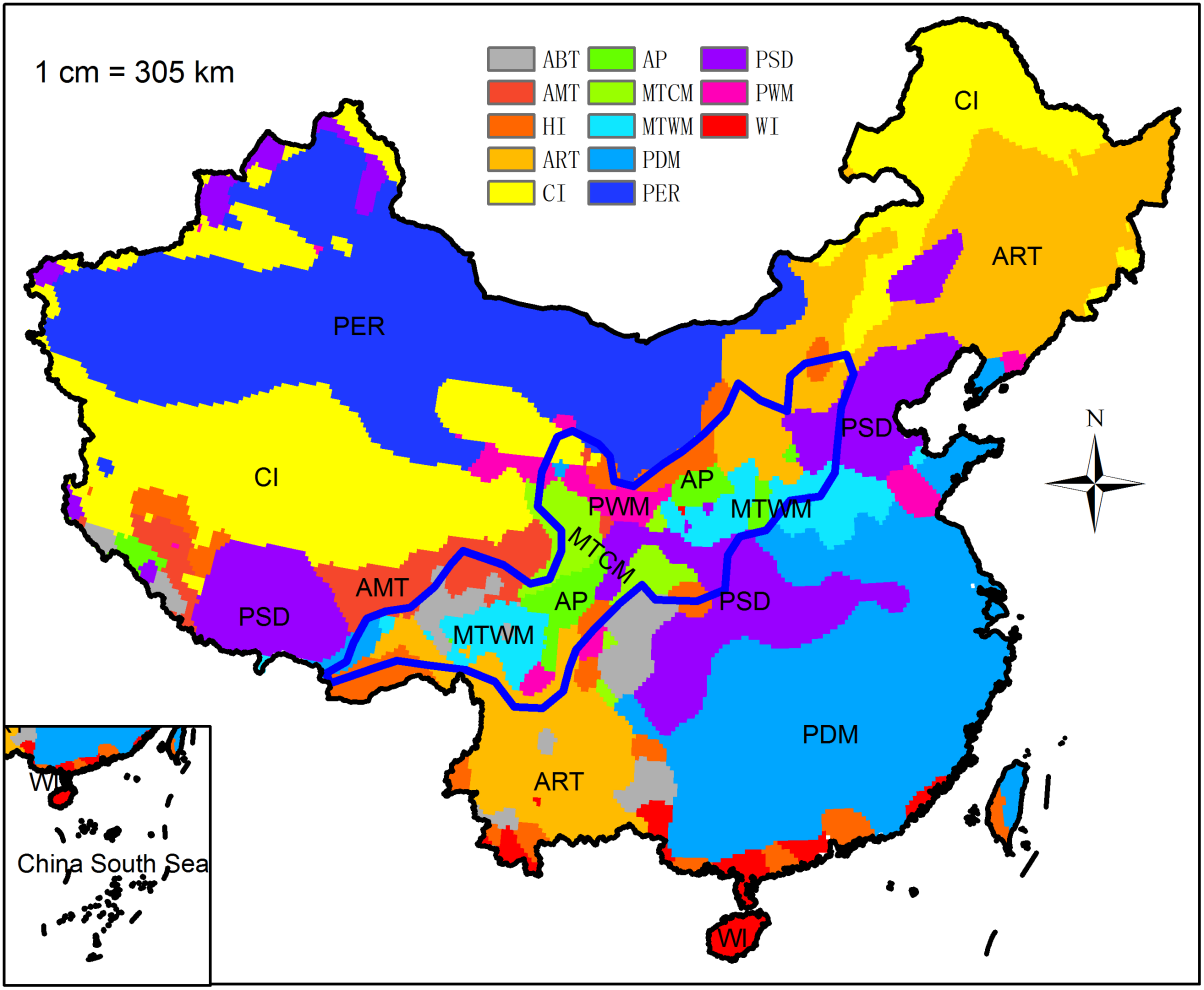
Spatial distribution of limiting factors for Chinese sea buckthorn in China. Coarse blue polygon represents potential distribution range of Chinese sea buckthorn using threshold of 0.5.

Evaluation of climatic factors revealed that the distribution of Chinese sea buckthorn is mainly affected by PDM, AP, CI and ART. These factors are associated with the strong, cold Siberian winds and high altitudes of the Tibetan Plateau, which demonstrate that winter climate conditions play an important role in determining the potential distribution of Chinese sea buckthorn. Chinese sea buckthorn is a drought-tolerant plant that requires little water in winter (Table 2 and Fig 5). The limiting factors mapping (Fig 8) shows that high rainfall is the limiting factor determining the southern boundary of the species, as shown in the response curves of PDM and AP. It is possible that excessive rainfall causes Chinese sea buckthorn to be out-competed by forest species, leading to the restriction of southern boundary. Low temperature is the limiting factor determining the northern boundary of this species, which is shown by the response curves of CI and ART. Low temperatures may limit plant physiological tolerance, leading to low survivability of Chinese sea buckthorn in the far northern areas.

A serious soil erosion problem exists within the range of potential distribution region of Chinese sea buckthorn. Chinese sea buckthorn is an effective nitrogen fixing plant with a well-developed root system, it has been widely planted for preventing soil erosion. At the same time, Chinese sea buckthorn is a plant species of high economic value (nutritious food, high medicine and firewood), and its potential distribution region is suitable for the construction of Chinese sea buckthorn farms, especially the core distribution area, which can improve the current cropping system and planting structure. We found that the climatic thresholds of the core area of Chinese sea buckthorn are 1.0–7.0 mm for PDM, 344.0–1089.0 mm for AP, -47.7–0.0°C for CI, and 26.1–45.0°C for ART, which were estimated based on the map of the climatically suitable habitats (Fig 2). Many studies have revealed that shifts in the climatic niche of plant species seldom occurred (niche conservative) [65–67]. Therefore, we can calculate the fitness of Chinese sea buckthorn under any given climatic conditions by using the climatic thresholds reported in Table 2 and the response curves shown in Fig 5 or S1 Fig. We could also use local weather station data to indicate when this species could be planted and cultivated.

It is worth mentioning that the predicted distribution map is based on statistical models MaxEnt, which does not currently consider soil factors and land use types. Thus, the predicted suitable area might partly be unsuitable for cultivation of the species (e.g. urban land, water body). This study mainly focused on the most suitable climatic habitat and the climatic limiting factors for Chinese sea buckthorn at a national scale with a 10 arc-min resolution. It is the first step of macro planning, and it is necessary to consider local site conditions when this predictive map is applied in practice. Future research in the predicted core suitable area should focus on experimental introduction and cultivation, breeding, selecting prior planting area, and integrating macro-planning of cropping systems and planting structure. Considering the potential economic and ecological value of Chinese sea buckthorn, the plant species would contribute greatly to controlling soil erosion and increasing the income of local farmers in the future.

## Conclusions

Chinese sea buckthorn is a plant species of high economic value (nutritious food, high medicine and firewood) and a plant species of effective nitrogen fixing with a well-developed root system. It has been widely planted for preventing soil erosion. For scientific cultivation of this species, there is a need to identify suitable habitat where the species is most optimal for survival. To realize this target, this study collected 97 occurrence records of Chinese sea buckthorn from herbarium and publications, and used 13 climatic variables to simulate potential suitable habitat for Chinese sea buckthorn using MaxEnt and GIS. The simulated potential distribution area of Chinese sea buckthorn is mainly located in semi-humid and semi-arid regions and begins from southwest of the Hengduanshan Mountains and extends to the southern boundary of the Daxinganling Mountains, which occupies 11.7% of the land area of China and spans 12 provinces including Xizang, Yunnan, Sichuan, Gansu, Qinghai, Ningxia, Shaanxi, Shanxi, Hebei, Inner Mongolia, Henan, and Liaoning with the core areas mainly in Xizang, Sichuan, Gansu, Qinghai, Ningxia and Shaanxi provinces. The northern boundary of this species is mainly limited by extreme cold temperate (CI and ART), and the southern boundary of this species is mainly affected by abundant rainfall (AP), especially in winter (PDM). We can calculate the fitness of Chinese sea buckthorn under any given climatic conditions by using the climatic thresholds reported in Table 2 and the response curves shown in Fig 5 or S1 Fig. We can also use local weather station data to indicate when this species can be planted and cultivated. This study mainly focuses on the most suitable climatic habitat and the climatic limiting factors for Chinese sea buckthorn at a national scale with a 10 arc-min resolution. Thus, the predicted suitable habitat might partly be unsuitable for cultivation of the species. It is the first step of macro planning, and it is necessary to consider local site conditions when this predictive map is applied in practice. We believe that identification of priority areas for planting of Chinese sea buckthorn can be realized by integrating remote sensing data and boundary of suitable habitat of the species.

## Supporting Information

S1 Fig The response curves of climatic habitat suitability values for all climatic factors and their relative importance showing in the upper-left corner of corresponding subplot. (TIF)

S1 File Latitude and longitude coordinates of 97 records for Chinese sea buckthorn. (XLS)

## References

[1] Lian YS, Chen XL. System classification of *Hippophae* spp. Hippophae. 1996; 9: 15–24.

[2] Delectis Florae Reipublicae Popularis Sinicae Agendae Academiae Sinicae Edita Flora Reipublicae Popularis Sinicae. Beijing: Science Press; 1994.

[3] Small E, Catling PM, Li TSC. Blossoming treasures of biodiversity: sea buckthorn (*Hippophae rhamnoides*)—an ancient crop with modern virtues. Biodiveristy. 1996; 3: 25–27.

[4] Xiong BQ, Yu D, Yuan J, Zeng M, Zhang Y, Du JB. The wild plant resources and utilization of *Hippophae* in China. Chinese Wild Plant Resour. 2004; 23: 25–26.

[5] Li TSC, Schroeder WR. Sea buckthorn (*Hippophae rhamnoides* L.): a multipurpose plant. HortTechnology. 1996; 6: 370–380.

[6] An BL, Lu SG. Conservation and utilization of germplasm resources of sea buckthorn. Global Seabuck Res Devel. 2004; 2: 12–15.

[7] Chen YM, Liu GB, Xu BC, Chen YQ. Research progress and prospect of function on soil and water conservation of sea buckthorn in China. Sci Soil Water Conserv. 2004; 2: 88–93.

[8] Holdridge LR. Determination of world plant formations from simple climatic data. Science. 1947; 105: 367–368. 17800882 10.1126/science.105.2727.367

[9] Woodward FI. Climate and plant distribution. Cambridge: Cambridge University Press; 1987.

[10] Pearson RG, Dawson TP. Predicting the impacts of climate change on the distribution of species: are bioclimate envelope models useful? Global Ecol Biogeogr. 2003; 12: 361–371.

[11] Guisan A, Tingley R, Baumgartner JB, Naujokaitis-Lewis I, Sutcliffe PR, Tulloch AI, et al Predicting species distributions for conservation decisions. Ecol Lett. 2013; 16: 1424–1435. 10.1111/ele.12189 24134332 PMC4280402

[12] Mckenzie D, Peterson DW, Peterson DL, Thornton PE. Climatic and biophysical controls on conifer species distribution in mountain forests of Washington State, USA. J Biogeogr. 2003; 30: 1093–1108.

[13] Toledo M, Poorter L, Pena-Claros M, Alarcon A, Balcazar J, Leano C, et al Climate is a stronger driver of tree and forest growth rates than soil and disturbance. J Ecol. 2011; 99: 254–264.

[14] Gu H, Zhang H, Chen C, Liu XB. Nature distribution of *Hippophae rhamnoides* L. subsp. *sinensis* Rousi. in Qinghai-Tibetan Plateau and its relationships with main environmental factors. Int Res Devel Seabuck. 2008; 6: 10–16.

[15] Lian YS, Chen XL. The ecogeographical distribution of *Hippophae rhamnoides* subsp. *sinensis* and its phytogeographical significance. Acta Phytotaxon Sin. 1992; 30: 349–355.

[16] Hizel AH, LeLay G. Habitat suitability modeling and niche theory. J Appl Ecol. 2008; 45: 1372–1381.

[17] Elith J, Leathwick JR. Species distribution models: ecological explanation and prediction across space and time. Annu Rev Ecol Evol S. 2009; 40: 677–697.

[18] Nori J, Urbina-Cardona JN, Loyola RD, Lescano JN, Leynaud GC. Climate change and American bullfrog invasion: what could we expect in South America? PLoS ONE. 2011; 6(10): e25718 10.1371/journal.pone.0025718 21991339 PMC3185029

[19] Li GQ, Liu CC, Liu YG, Yang J, Zhang XS, Guo K. Advances in theotetical issues of species distribution models. Acta Ecol Sin. 2013; 33: 4827–4835.

[20] Wisz MS, Hijmans RJ, Li J, Peterson AT, Graham CH, Guisan A, et al Effects of sample size on the performance of species distribution models. Divers Distrib. 2008; 14: 763–773.

[21] Elith J, Graham CH, Anderson RP, Dudik M, Ferrier S, Guisan A, et al Nevel methods improve prediction of species's distribution from occurrence data. Ecography. 2006; 29: 129–151.

[22] Franklin J. Mapping species distributions: spatial interence and prediction. Cambridge: Cambridge University Press; 2009.

[23] Peterson AT, Soberon J, Pearson RG, Andersen RP, Martinez-Meyer E, Nakamura M, et al Ecological niches and geographic distributions—monographsin population biology No. 49. Princeton: Princeton University Press; 2011.

[24] Pattison RR, Mack RN. Potential distribution of the invasive tree *Triadica sebifera* (Euphorbiaceae) in the United States: evaluating CLIMEX predictions with field trials. Global Change Biol. 2008; 14: 813–826.

[25] Poutsma J, Loomans AJM, Aukema B, Heijerman T. Predicting the potential geographical distribution of the *Harlequin ladybird*, *Harmonia axyridis*, using the CLIMEX model. Biocontrol. 2008; 53: 103–125.

[26] Shabani F, Kumar L. Risk levels of invasive fusarium oxysporum f. sp in areas suitable for Date Palm (*Phoenix dactylifera*) cultivation under various climate change projections. PLoS ONE. 2013; 8(12): e83404 10.1371/journal.pone.0083404 24340100 PMC3858343

[27] Shabani F, Kumar L, Taylor S. Climate change impacts on the future distribution of Date Palms: a modeling exercise using CLIMEX. PLoS ONE. 2012; 7(10): e48021 10.1371/journal.pone.0048021 23110162 PMC3480471

[28] Taylor S, Kumar L, Reid N, Kriticos DJ. Climate change and the potential distribution of an invasive shrub, *Lantana camara* L. PLoS ONE. 2012; 7(4): e35565 10.1371/journal.pone.0035565 22536408 PMC3334920

[29] Yonow T, Sutherst RW. The geographical distribution of the Queensland fruit fly, *Bactrocera (Dacus) tryoni*, in relation to climate. Aust J Agr Res. 1998; 49: 935–953.

[30] Nazeri M, Kumar L, Jusoff K, Bahaman AR. Modeling the potential distribution of Sun Bear in Krau Wildlife Reserve, Malaysia. Ecol Inform. 2014; 20: 27–32.

[31] Gormley AM, Forsyth DM, Griffioen P, Lindeman M, Ramsey DS, Scroggie MP, Woodford L. Using presence-only and presence-absence data to estimate the current and potential distributions of established invasive species. J Appl Ecol. 2011; 48: 25–34. 21339812 10.1111/j.1365-2664.2010.01911.x PMC3038347

[32] Stockwell D, Peters D. The GARP modelling system: problems and solutions to automated spatial prediction. Int J Geogr Inf Sci. 1999; 13: 143–158.

[33] Phillips SJ, Anderson RP, Schapire RE. Maximum entropy modeling of species geographic distributions. Ecol Model. 2006; 190: 231–259.

[34] Phillips SJ, Elith J. On estimating probability of presence from use-availability or presence-background data. Ecology. 2013; 94: 1409–1419. 23923504 10.1890/12-1520.1

[35] Booth TH, Nix HA, Busby JR, Hutchinson MF. BIOCLIM: the first species distribution modelling package, its early applications and relevance to most current MAXENT studies. Divers Distrib. 2014; 20: 1–9.

[36] Carpenter G, Gillison AN, Winter J. Domain: a flexible modelling procedure for mapping potential distributions of plants and animals. Biodivers Conserv. 1993; 2: 667–680.

[37] Nazeri M, Jusoff K, Madani N, Mahmud AR, Bahman AR, Kumar L. Predictive modeling and mapping of Malayan Sun Bear (*Helarctos malayanus*) distribution using maximum entropy. PLoS ONE. 2012; 7(10): e48104 10.1371/journal.pone.0048104 23110182 PMC3480464

[38] Navarro-Cerrillo RM, Hernandez-Bermejo JE, Hernandez-Clemente R. Evaluating models to assess the distribution of *Buxus balearica* in southern Spain. Appl Veg Sci. 2011; 14: 256–267.

[39] Elith J, Kearney M, Phillips S. The art of modelling range-shifting species. Methods Ecol Evol. 2010; 1: 330–342.

[40] Hou XY. Vegetation of China with reference to its geographical distribution. Ann Mo Bot Gard. 1983; 70: 509–549.

[41] Chen XL, Lian YS. Distribution pattern of *Hippophae* spp. and its cause. Hippophae. 2007; 20: 1–5.

[42] CVH. Chinese Virtual Herbarium. 10 May 2014. Accessed: http://www.cvh.org.cn/.

[43] Worldclim. Global Climate Data—Free climate data for ecological modeling and GIS. 10 May 2014. Accessed: http://wwwworldclimorg/.

[44] Hijmans RJ, Cameron SE, Parra JL, Jones PG, Jarvis A. Very high resolution interpolated climate surfaces for global land areas. Int J Climat. 2005; 25: 1965–1978.

[45] Kira T. A new classification of climate in Eastern Asia as the basis for agricultural geography. Kyoto: Horticultural institute, Kyoto University; 1945.

[46] Xu WD. Kira's temperature indices and their application in the study of vegetation. Chinese J Ecol. 1985: 35–39.

[47] Li GQ, Xu GH, Guo K, Du S. Mapping the global potential geographical distribution of black locust (*Robinia pseudoacacia* L.) using herbarium data and a maximum entropy model. Forests. 2014; 5: 2773–2792.

[48] Phillips SJ, Dudik M, Schapire RE. A maximum entropy approach to species distribution modeling In Proceedings of the Twenty-First International Conference on Machine Learning, South Banff, Alberta, Canada; 4–8 7 2004 pp. 655–662.

[49] Fielding AH, Bell JF. A review of methods for the assessment of prediction errors in conservation presence/absence models. Environ Conserv. 1997; 24: 38–49.

[50] Swets KA. Measuring the accuracy of diagnostic system. Science. 1988; 240: 1285–1293. 3287615 10.1126/science.3287615

[51] ESRI. ArcGIS 9.3, Redlands, California, USA. 2012.

[52] Editorial Committee for Vegetation Atlas of China. 1:100 million Vegetation Atlas of China. Beijing: Science Press; 2001.

[53] Macfadyen S, Kriticos DJ. Modelling the geographical range of a species with variable life-history. PLoS ONE. 2012; 7(7): e40313 10.1371/journal.pone.0040313 22808133 PMC3394791

[54] Taylor S, Kumar L, Reid N. Impacts of climate change and land-use on the potential distribution of an invasive weed: a case study of *Lantana camara* in Australia. Weed Res. 2012; 52: 391–401.

[55] Taylor S, Kumar L. Potential distribution of an invasive species under climate change scenarios using CLIMEX and soil drainage: A case study of *Lantana camara* L. in Queensland, Australia. J Environ Manage. 2013; 114: 414–422. 10.1016/j.jenvman.2012.10.039 23164541

[56] Gaston A, Garcia-Vinas JI, Bravo-Fernandez AJ, Lopez-Leiva C, Oliet JA, Roig S, et al Species distribution models applied to plant species selection in forest restoration: are model predictions comparable to expert opinion? New Forest. 2014; 45: 641–653.

[57] Hodder DP, Johnson CJ, Rea RV, Zedrosser A. Application of a species distribution model to identify and manage bear den habitat in central British Columbia, Canada. Wildlife Biol. 2014; 20: 238–245.

[58] Guisan A, Rahbek C. SESAM—a new framework integrating macroecological and species distribution models for predicting spatio-temporal patterns of species assemblages. J Biogeogr. 2011; 38: 1433–1444.

[59] Brambilla M, Ficetola GF. Species distribution models as a tool to estimate reproductive parameters: a case study with a passerine bird species. J Anim Ecol. 2012; 81: 781–787. 10.1111/j.1365-2656.2012.01970.x 22372863

[60] Editorial Committee for Physical Geography of China. Physical geography of China. Beijing: Science Press; 1985.

[61] Guo K, Werger MJA. Effect of prevailing monsoons on the distribution of beeches in continental East Asia. Forest Ecol Manag. 2010; 259: 2197–2203.

[62] Liu HY, Yin Y, Wang QY, He SY. Climatic effects on plant species distribution within the forest-steppe ecotone in northern China. Appl Veg Sci. 2015; 18: 43–49.

[63] Liu HY, Cui HT, Yu PT, Huang YM. The origin of remnant forest stands of *Pinus tabulaeformis* in southeastern Inner Mongolia. Plant Ecol. 2002; 158: 139–151.

[64] Li GQ, Liu CC, Liu YG, Yang J, Zhang XS, Guo K. Effects of climate, disturbance and soil factors on the potential distribution of Liaotung oak (*Quercus wutaishanica* Mayr) in China. Ecol Res. 2012; 27: 427–436.

[65] Petitpierre B, Kueffer C, Broennimann O, Randin C, Daehler C, Guisan A. Climatic niche shifts are rare among terrestrial plant invaders. Science. 2012; 335: 1344–1348. 10.1126/science.1215933 22422981

[66] Booth TH, Nix HA, Hutchinson MF, Jovanovic T. Niche analysis and tree species introduction. For Ecol Manag. 1988; 23: 47–59.

[67] Wellenreuther M, Larson KW, Svensson EI. Climatic niche divergence or conservatism? Environmental niches and range limits in ecologically similar damselflies. Ecology. 2012; 93: 1353–1366. 22834376 10.1890/11-1181.1

