# Correction: Evaluation of Limiting Climatic Factors and Simulation of a Climatically Suitable Habitat for Chinese Sea Buckthorn

**DOI:** 10.1371/journal.pone.0136001

**Published:** 2015-08-13

**Authors:** Guoqing Li, Sheng Du, Ke Guo

The images for Figs 6 and 7 are incorrectly switched. The image that appears as Fig 6 should be Fig 7, and the image that appears as Fig 7 should be Fig 6. The figure captions appear in the correct order. Please see the correct Figs 6 and 7 here.

**Fig 6:**
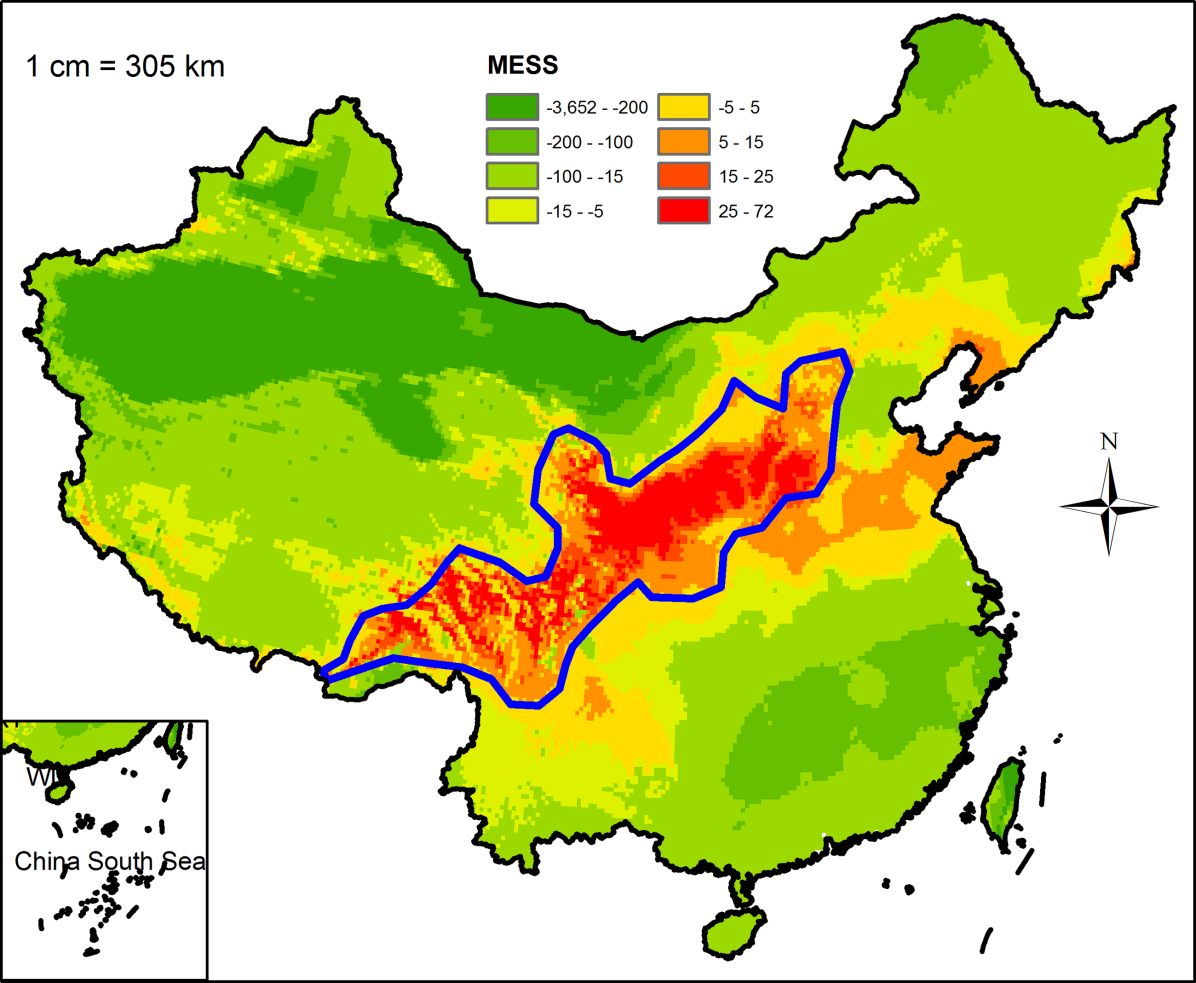
Multivariate environmental similarity surface (MESS) map of novel habitat. Coarse blue polygon represents potential distribution range of Chinese sea buckthorn using threshold of 0.5. Red color represents interpolation habitat (positive value), green color represents extrapolation habitat (negative value), and orange represents marginal habitat (near zero).

**Fig 7:**
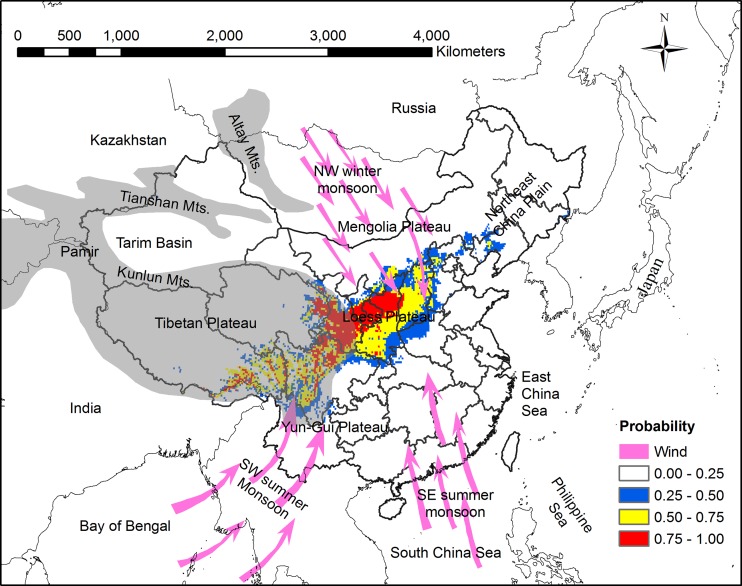
Distribution pattern of Chinese sea buckthorn, monsoon climate and four Plateaus (Tibetan Plateau, Yun-Gui Plateau, Loess Plateau, and Mengolia Plateau) in China. Grey portion represents high altitude region including Tibetan Plateau, Pamir Plateau, Tianshan Mts., and Altay Mts. Monsoon climate includes the northwest winter monsoon, the southwest summer monsoon, and the southeast summer monsoon.
